# Genotypic Characterization of *Mycobacterium bovis* Isolates From Dairy Cattle Diagnosed With Clinical Tuberculosis

**DOI:** 10.3389/fvets.2021.747226

**Published:** 2021-10-11

**Authors:** Elizabeth Hortêncio de Melo, Harrison Magdinier Gomes, Philip Noel Suffys, Márcia Quinhones Pires Lopes, Raquel Lima de Figueiredo Teixeira, Ícaro Rodrigues dos Santos, Marília Masello Junqueira Franco, Helio Langoni, Antonio Carlos Paes, José Augusto Bastos Afonso, Carla Lopes de Mendonça

**Affiliations:** ^1^Programa de Pós-Graduação em Medicina Veterinária, Universidade Federal Rural de Pernambuco, Recife, Brazil; ^2^Laboratório de Biologia Molecular Aplicada à Micobactérias, Fundação Oswaldo Cruz, Laboratório de Biologia Molecular Aplicada à Micobactérias, Rio de Janeiro, Brazil; ^3^Departamento de Higiene Veterinária e Saúde Pública, Faculdade de Medicina Veterinária e Zootecnia, Universidade Estadual Paulista, Botucatu, Brazil; ^4^Clínica de Bovinos de Garanhuns, Universidade Federal Rural de Pernambuco, Garanhuns, Brazil

**Keywords:** bovine tuberculosis, dairy cattle, genotyping, *Mycobacterium bovis*, pathology, spoligotyping, MIRU-VNTR

## Abstract

Molecular diagnosis of bovine tuberculosis plays an essential role in the epidemiological knowledge of the disease. Bovine tuberculosis caused by *Mycobacterium bovis* represents a risk to human health. This study aimed to perform the genotypic characterization of *M. bovis* isolated from bovines diagnosed as tuberculosis from dairy herds in the state of Pernambuco, Brazil. Granulomas from 30 bovines were sent for microbiological culture, and colonies compatible with *Mycobacterium* spp. were obtained in at least one culture from 17/30 granulomas. All isolates were confirmed to be *M. bovis* by *spoligotyping* and 24*loci* MIRU-VNTR typing. While *spoligotyping* characterized the isolates as SB0121, SB0295, SB0852, SB0120, and an unclassified genotype, 24*loci* MIRU-VNTR rendered two clusters of two isolates each and 13 unique profiles. *Loci* ETR-A showed higher discriminatory power, and *loci* (ETR-B, ETR-C, MIRU16, MIRU27, and QUB26) showed moderate allelic diversity. This is the first study on the genetic variability of the infectious agent cause of bovine TB in Pernambuco and demonstrates variability of strains in the state. Thus, it corroborates the importance of this microorganism as agent of bovine tuberculosis and its zoonotic potential, this epidemiological tool being a determinant in the rigor of the sanitary practices of disease control in dairy herds.

## Introduction

Bovine tuberculosis is a chronic progressive disease caused by *Mycobacterium bovis* which affects mainly cattle and buffalo but also infects other mammalian species of mammals, including humans (1). The zoonotic potential of this disease is related to the consumption of raw milk and unpasteurized derivatives, representing the main route of transmission to humans, more pronounced in rural areas. In the state of Pernambuco, a prevalence of outbreaks of 2.87 and 0.62% of infected animals was reported in 2016, with a tendency to concentrate in the Agreste region of the state and with a predominance in dairy properties (2).

The interest in nucleic acid-based diagnostic procedures increased because of the limitations of conventional testing such as lack of sensitivity and specificity of the allergic-skin test and the long period for confirming the presence of the agent by bacteriological methods (3). In addition, molecular typing methods have provided a great impetus in the molecular epidemiology studies of the *M. tuberculosis* complex including comparing mycobacterial genome sequences. Among the most used genotyping techniques for the study of the *M. tuberculosis* complex are *Spoligotyping* and Variable Number of Interspersed Repetitive Units of Mycobacteria (MIRU-VNTR) (4, 5). MIRU-VNTR has higher discriminatory power and has currently been the method of choice in the genotyping studies of *Mycobacterium* spp. and, in particular related to *M. bovis*, allows the identification of prevalent strains circulating in a herd or geographic regions (4, 5).

*M. bovis* infection has an impact on both animal and human health; non-etheless, scarce are the studies in the region on molecular genotyping. Given the lack of data on the contribution and nature of the *Mycobacterium tuberculosis* complex (MTBC) to bovine TB in the state of Pernambuco, we performed the genotypic characterization of Mycobacteria isolated from bovines from dairy herds in this region that were diagnosed clinically with tuberculosis, coming from dairy herds in the state of Pernambuco.

## Materials and Methods

The study included 28 bovines and two buffaloes that had been attended at the Bovine Clinic of Garanhuns/UFRPE, presenting clinical symptoms suggestive for tuberculosis. The animals were submitted to clinical examination, with information, including epidemiological, that was annotated in clinical records. Among the information present in the anamnesis provided by the owners, common to most animals, were progressive weight loss, dry cough, and decreased milk production.

According to the evolution/severity of the clinical cases and the result of the allergic-skin test, the animals were euthanized according to the current legislation (Brazil, Ministry of Agriculture, Livestock and Supply. Normative Instruction n. 19, 10 of October, 2016) and submitted for anatomopathological examination.

Fragments of organs with lesions characteristic of granulomas were collected for histopathological examination and lymph nodes with lesions for microbiological culture. The samples for bacteriology were stored in a freezer (−80°C) for further processing, while for histopathological evaluation, fragments were fixed in 10% buffered formaldehyde, processed, and stained with hematoxylin and eosin (HE). Granulomas from all 30 animals were collected and sent for microbiological culture and sample processing, and culture conditions favoring isolation of *M. bovis* were carried out following the recommendations of Franco et al. (6). Samples were minced and decontaminated according to the Petroff method, inoculated on Löwenstein–Jensen and Stonebrink medium, and incubated at 37°C for 90 days.

Nucleic acid was obtained from the cells by thermolysis. Molecular identification to the *Mycobacterium* species was performed by PCR amplification of a 1,020-bp fragment of the *gyr*B gene, as described by Chimara et al. (7) and Franco et al. (6). In the reaction, 1 μl of DNA (20 ng) and 47 μl of Master Mix (1 ×) were used (Thermo Scientific, Waltham, MA, USA), as well as 10 pM of each of the primers MTUBf (5′ TCGGACGCGTATGCGATATC 3′) and MTUBr (5′ ACATACAGTTCGGACTTGCG 3′) [DNA Express Biotecnologia LTDA, Brazil]. The cycling profile consisted of denaturation at 95°C for 10 min, followed by 35 amplification cycles at 94°C for 1 min, 65°C for 1 min, and 72°C for 1.5 min, and a final extension at 72°C for 10 min. The amplification and fragment size were confirmed by electrophoresis in agarose gel (1%) stained with GelRed™ (Biotium, Hayward, CA, USA) using a 100-bp molecular marker (DNA Express Biotecnologia LTDA). Then, 10 μl of the amplified product was submitted for restriction fragment length polymorphism (RFLP) through digestion by restriction enzymes *Rsa*I, *Taq*I, and *Sac*II (Thermo Scientific, Waltham, MA, USA), following the manufacturer's recommendations. The generated fragments were separated on 2% agarose gel stained with GelRed™ using 50- and 100-bp molecular markers (DNA Express Biotecnologia LTDA). After electrophoresis, the gels were photographed in photo-documentation equipment (2UV Transilluminator UVP) and restriction patterns compared to those described by Chimara et al. (7).

*Spoligotyping* was performed as described by Kamerbeek et al. (4), and the amplified products underwent membrane hybridization (manufacturing *in-house*) with 43 oligonucleotides. For amplification of the DR region, 20 μM of each of primers DRa 5′ GGTTTTGGGTCTGACGAC 3′ (5′ biotinylated) and DRb (5′ CCGAGAGGGGACGGAAAC 3′), MyTaq Mix (12.5 μl), 1 μl (20 ng) genomic DNA, and ultra-pure water (9.5 μl) were submitted to PCR in a final volume of 25 μl.

MIRU-VNTR typing using a combination of 24-*loci* was performed according to Supply et al. (5). In each PCR reaction, 10 μl MyTaq Mix (Bioline®), 0.4 μl of each *primer* (20 mM), 2 μl of DNA (20 ng), and 7.2 μl of ultra-pure water were used in the final volume of 20 μl. *Mycobacterium tuberculosis* H37Rv DNA and water were used as positive and negative controls, respectively.

The genetic profile based on *spoligotyping* of each isolate was compared to those present in the international databases http://www.mbovis.org/ and http://www.pasteur-guadeloupe.fr:8081/SITVITONLINE. The 24-MIRU-VNTR patterns were compared to those present in the MIRU-VNTRplus database deposited in the application: http://www.miru-vntrplus.org/MIRU/index. The Hunter–Gaston discriminatory index (HGDI) was performed to evaluate the variability of the genotypes obtained by spoligotyping, and each of the alleles of 24-MIRU-VNTR typing.

## Results

The 17 animals from which *M. bovis* was isolated came from 10 municipalities in the state of Pernambuco (Alagoinha, Bom Conselho, Chã Grande, Garanhuns, Ibirajuba, Jurema, Pedra, Pesqueira, Ribeirão, and Venturosa), which were mostly raised in the semi-intensive management system. These municipalities belong to three geographic regions of the state, namely, Southern Agreste, Central Agreste, and South Agreste. Among the animals diagnosed with the disease, females were the most affected (16/17) and 64.7% (11/17) were older than 5 years; one calf 7 months old also yielded positive culture.

The clinical examination of cattle and buffaloes revealed apathy, lack of appetite, low body mass score, seromucous nasal discharge, dry cough, dyspnea, tachypnea, polyps, crackles, and areas of silence in the lung fields. Upon evaluation of the mammary gland, two (2/17) bovines were diagnosed with hypertrophied lymph nodes: one of these presented an enlarged posterior breast of firm consistency, hyperemia and hyperthermia, and physical changes in milk in one of the teats (lumps with serum). The other bovine had an anterior breast of firm consistency but with no visible changes of the milk. During rectal examination, some animals presented nodular structures of varying sizes and hardened consistency in the region of the mesentery, serous in the rumen, and uterus.

Macroscopic observation of lesions seen during *postmortem* examination revealed that 12/17 animals (70.6%) had miliary or protruding tuberculosis, distributed mainly in the lungs, mediastinal and tracheobronchial lymph nodes, liver, and mesenteric lymph nodes and less frequently in the kidneys, spleen, and greater omentum. Among the animals with generalized tuberculosis, two cattle also showed changes in the mammary gland and the uterus, characterized by granulomatous lesions with multifocal distribution and varied sizes, with areas of calcification and abscesses.

**Figure 1:**
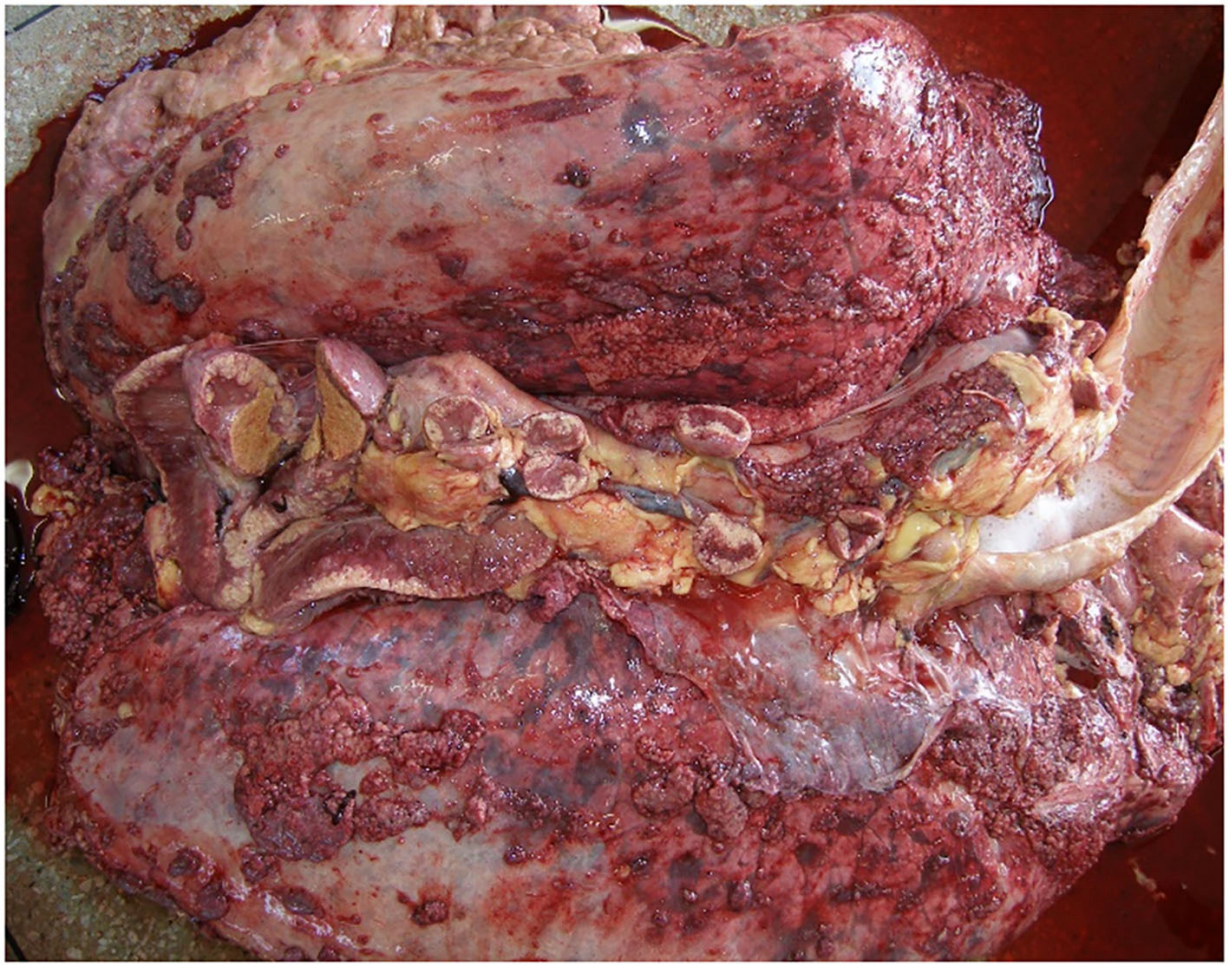
Granulomatous lesions distributed in lung and mediastinal lymph nodes of bovines.

**Figure 2:**
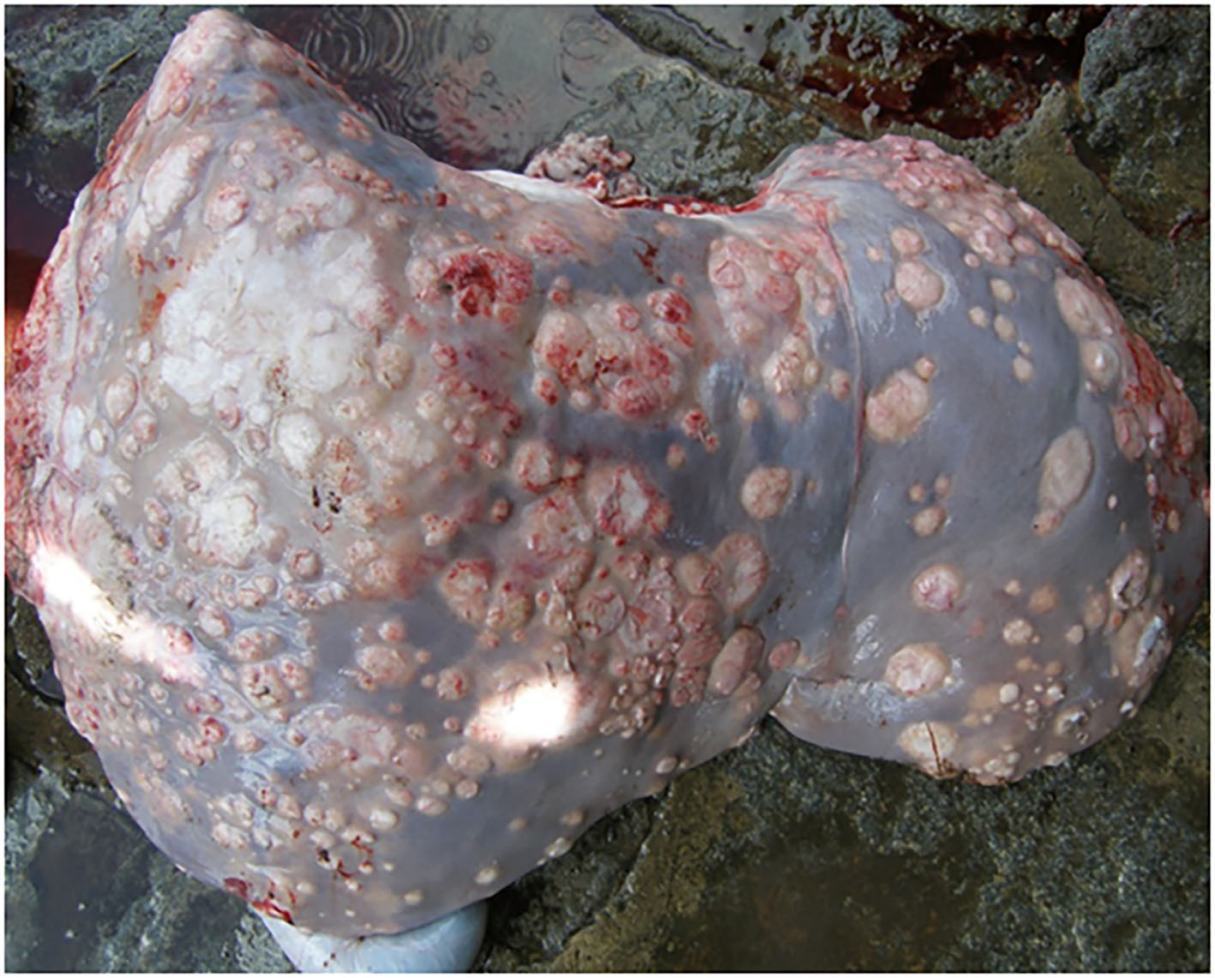
Granulomatous lesions distributed in the liver of buffaloes.

The granulomatous nodules observed in all animals were pleomorphic and had a caseous, thick, and yellowish content, with the formation of a fibrous capsule (Figure 1). In buffaloes, granulomas had a more whitish color when compared to cattle (Figure 2). In the young calf, in addition to lung lesions, small granulomas were observed in the central nervous system and lesions compatible with meningoencephalitis.

Histopathological analysis of the lesions revealed areas of central caseous necrosis and dystrophic calcification and intense inflammatory reaction in the regions adjacent to the necrosis areas, with a predominance of epithelioid macrophages and multinucleated giant cells, like *Langhans*.

Microbiological cultivation presented growth of colonies in 17/30 (57%) samples that were confirmed to be *Mycobacterium* spp. and more specifically *M. bovis* by molecular techniques. In three samples, presence of *Trueperella pyogenes* and, in a single animal*, Nocardia* spp. was encountered. Of the 17 bacterial growths, 14 were classified by the enzymatic restriction analysis of the *gyr*B gene as *M. bovis*. However, due to the importance of bacterial isolation, recognized as a gold standard test, the 17 samples were submitted to molecular genotyping techniques by *Spoligotyping* and 24-*loci* MIRU-VNTR.

*Spoligotyping* revealed five spoligotypes classified as belonging to *M. bovis*, including SB0121, SB0295, SB0852, SB0120, and a *spoligotype* that was not yet present in the database (Table 1).

**Table 1:** Molecular characterization of *M. bovis* isolates from cattle in the state of pernambuco by *Spoligotyping* and MIRU-VNTR.

**ID**	**Octal spoligotype**	**Cluster spoligotype**	**Profile of 24 MIRU-VNTR**	**Cluster MIRU-VNTR**	**SIT**	**Mbovis.org**	**Municipality**
**1**	676773677777600	Cluster S1	2^*^63^***^313^*^3434253332^*^12	Orphan	481	SB0121	Bom conselho
**5**	676773677777600	Cluster S1	226322332324414253332622	Orphan	481	SB0121	Garanhuns
**6**	676773677777600	Cluster S1	226322332354434253332622	Orphan	481	SB0121	Chã grande
**7**	676773677777600	Cluster S1	22^*^322432364434253332632	Orphan	481	SB0121	Alagoinha
**9**	676773677777600	Cluster S1	^*^2^*^322332363444253332312	Orphan	481	SB0121	Pedra
**10**	676773677777600	Cluster S1	2^*^5^******^3^*^34^*****^3^*^2^***^	Orphan	481	SB0121	Pesqueira
**15**	676773677777600	Cluster S1	^**^5322^*^32353434253332^*^12	Orphan	481	SB0121	Bom conselho
**26**	676773677777600	Cluster S1	225322232353444253332512	Orphan	481	SB0121	Pesqueira
**29**	676773677777200	Cluster S2	224322232353444253332112	Orphan	698	SB0295	Jurema
**34**	676773677777200	Cluster S2	225322232353444251332512	Orphan	698	SB0295	Jurema
**38**	676773677777200	Cluster S2	2^*^5322332343444253332612	Orphan	698	SB0295	Ibirajuba
**39**	676773677777200	Cluster S2	2^*^4322332343444251332512	Cluster M1	698	SB0295	Ribeirão
**40**	676773677777200	Cluster S2	2^*^432233234^*^444251332512	Cluster M1	698	SB0295	Ribeirão
**35**	676773777777200	Cluster S3	225322132323434253333512	Cluster M2	797	SB0852	Bom conselho
**37**	676773777777200	Cluster S3	2^*^5322132323434253333512	Cluster M2	797	SB0852	Bom conselho
**12**	676773777777600	Orphan	^*^1^*^32231342^*^434251332512	Orphan	482	SB0120	Garanhuns
**2**	New profile	Orphan	23^*^322331553414243332612	Orphan	New	New	Venturosa

*Cluster S – cluster Spoligotyping Cluster M – cluster MIRU-VNTR*.

** *Isolates that presented amplification failures in some loci*.

*Order of the 24 loci of MIRU-VNTR patterns, MIRU 02–154; Mtub 04–424; ETR C−577; MIRU 04–580; MIRU 40–802; MIRU 10–960; MIRU 16–1,644; Mtub 21–1,955; MIRU 20–2059; QUB 11b-2163b; ETR A−2165; Mtub 29–2,347; Mtub 30–2,401; ETR B−2,461; MIRU 23–2,531; MIRU 24–2,687; MIRU 26–2,996; MIRU 27–3,007; Mtub 34–3,171; MIRU 31–3,192; Mtub 39 – 3,690; QUB 26 – 4052; QUB 4,156–4,156; MIRU 3–4,348*.

The analysis of 24-*loci* MIRU-VNTR identified 13 genetic profiles from the 17 isolates of *M. bovis* from 14 properties in the state of Pernambuco (Table 1).

The analysis of the discriminatory power (HGDI) of MIRU-VNTR in this study was higher, as expected, than *Spoligotyping*, respectively 0.980 and 0.713. Distribution of the isolates according to the number of alleles in each locus and the analysis of the allelic diversity of the 24-*loci* is summarized in Table 2. *Locus* ETR A showed the highest discriminatory power (*h* = 0.69), while five *loci* (ETR B, MIRU 16, ETR C, MIRU 27, and QUB 26) were classified as moderately discriminatory with *h* between 0.33 and 0.58. Eight *loci* (MIRU 20, MIRU 26, Mtub 04, Mtub 29, QUB 11b, QUB 4156, Mtub 21, Mtub 39) presented low discriminatory power (*h* ≤ 0.27) while 10 *loci* showed absence of allelic diversity.

**Table 2:** Distribution and allele diversity (HGDI) of the 24-*loci* MIRU-VNTR.

***Locus***	**Number of repetitions**						**Allele diversity HGDI**
	**1**	**2**	**3**	**4**	**5**	**6**	
MIRU 02		14					0.000
Mtub 04	1	8	1				0.266
ETR C				3	7	3	0.570
MIRU 04			16				0.000
MIRU 40		15					0.000
MIRU 10		15					0.000
MIRU 16	2	3	8	1			0.571
Mtub 21	1		15				0.058
MIRU 20	2	13	1				0.275
QUB 11b			15	1	1		0.165
ETR A		4		3	6	2	0.690
Mtub 29			12	3			0.271
Mtub 30				17			0.000
ETR B	2		7	7			0.575
MIRU 23				16			0.000
MIRU 24		16					0.000
MIRU 26				1	15		0.058
MIRU 27	4		12				0.333
Mtub 34			17				0.000
MIRU 31			16				0.000
Mtub 39		15	2				0.158
QUB 26	1		1		7	5	0.582
QUB 4,156	13	2	1				0.275
MIRU 39		16					0.000

Isolates one and 10 showed failures in the amplification of some *loci* that are generally attributed to possible DNA mutations or degradation (5), thus preventing the *primers* from ringing. Given these results, the respective isolates started to be analyzed only in *Spoligotyping*, obtaining significant results.

## Discussion

It should be noted that the state of Pernambuco occupies a prominent place in milk production in the Northeast region, and the municipality of Garanhuns and its microregion are recognized as the state's milk basin (8). Dairy cattle and buffaloes are considered more vulnerable to *M. bovis* infection, as they have a longer life expectancy, stay longer on the properties, and are subjected to the rearing semi-intensive and intensive systems, very common in the region. During milking and other common management practices, animals cohabit, therefore increasing their likelihood of contact and the transmission of tuberculosis (2, 9), considered endemic in the State of Pernambuco (2, 10). The constant transit of animals between the properties within and between neighboring municipalities, the interstate cattle trade, and the absence of an effective sanitary control of the herds are factors that contribute to the spread of the disease in the region (2, 9).

In the present study, all animals presented clinical symptoms of tuberculosis with predominating respiratory impairment. In dairy farms, female animals generally remain for longer periods depending on the reproductive period, and this could be the main reason for having observed in this study the predominance of females over the age of 5 years to be exposed to *M. bovis* when compared to young cattle (9). Non-etheless, young animals also contract the infection and develop disease, as demonstrated by *M. bovis* isolation from a 7-month-old calf. The frequency of tuberculosis in cattle aged <12 months is generally associated with the ingestion of colostrum/milk from infected cows or transplacental infection (11, 12). The most evident clinical signs were observed in the advanced stages of the disease, as described by Izael et al. (10) and Waters (13), except for the calf that manifested the disease earlier in the form of cerebral tuberculosis combined with depression and paresis of the limbs. In addition to the predominant respiratory impairment in the animals in this study, two animals showed clinical changes in the mammary gland, resulting to be similar to that described by Waters (13). This observation reinforces the potential risk of the disease to public health due to the consumption of raw milk and non-pasteurized derivatives, mainly observed in inland cities and rural areas, such as Garanhuns and the microregion (8).

The generalized form of the disease was predominant both in cattle and in the two buffaloes, with lesions that had disseminated to several organs. All animals had granulomatous injuries in the thoracic organs (lungs, pleura, tracheobronchial, and mediastinal lymph nodes), causing respiratory impairment. This result is similar to those described by Ramos et al. (14), who reported a higher prevalence of lesions compatible with tuberculosis in tracheobronchial and mediastinal lymph nodes and lungs; such typical predominance of lesions in the respiratory tract is indicative for airborne transmission. On the other hand, Alzamora Filho et al. (15) identified the most evident lesions in the lymph nodes of the head (retropharyngeal and parotid) with pulmonary parenchyma. These results corroborate with the findings of the present study, due to the typical predominance of lesions in the respiratory tract, suggesting the airway, as the main gateway for *M. bovis* in bovines. The lower occurrence of mesenteric lymph node involvement here observed was also described by Ramos et al. (14) and justified by the fact that oral route infection is secondary to the respiratory route in adult cattle.

The granulomatous lesions observed in the mammary gland and uterus common to two animals in this study reinforce the potential risk of transmission of *M. bovis* to humans due to the consumption of raw milk and its products (16, 17). On the other hand, the granulomatous lesions located in the central nervous system in young cattle are probably related to the ingestion of colostrum/milk from infected cows and can be justified by ascending infection *via* hematogenic route. This form of cerebral tuberculosis in cattle was also reported by Konradt et al. (11) and Silveira et al. (12).

The histopathological characterization of lesions present in granulomas was similar to the findings described by França et al. (18) who found in some samples a marked process of calcification with mineralization, differing from the lesions observed by Ramos et al. (14) and Silva et al. (19) who presented a more caseous aspect, suggesting that the animals that had been slaughtered were suffering from a recent infection or disease development.

The frequency of isolation of *M. bovis*, of 57%, was observed presently in animals, with clinical tuberculosis. It has been described that some factors can interfere with the success of mycobacterial isolation and in particular of *M. bovis*, including the rigorous decontamination process of samples and the chronic character of the disease that confers intense calcification of the lesions, leading to low concentrations or absence of viable bacilli (20). This might have been influenced by the low isolation of *M. bovis* in the present sampling.

Besides *Mycobacterium* spp., we also observed bacteria belonging to other genera such as *Trueperella pyogenes* and *Nocardia* spp. It is worth mentioning that some microorganisms besides these, such as *Actinomyces* spp. and *Actinobacillus* spp., are also responsible for causing granulomatous lesions similar to tuberculosis lesions (21).

In the present study, 17 isolates compatible with *Mycobacterium* spp. were subjected to molecular diagnostics by RFLP of the *gyr*B gene. However, the analysis classified only 14/17 isolates as *M. bovis*, different from the study carried out by Franco et al. (6) that obtained 100% compatibility between the isolation of *Mycobacterium* spp. and the *gyr*B analysis. The result obtained in the RFLP is probably related to factors that interfere with molecular tests, such as the presence of inhibitors of PCR reactions, low amount of viable bacilli due to chronic lesions, contaminants in the samples, and failures in extraction processing or DNA degradation (22).

*Spoligotype* SB0121, the most frequently encountered, was described as the most prevalent in national territory with a frequency of 29.1% in a study conducted in Latin American countries (23). The fact that we identified this *spoligotype* in the three defined geographical region studies here could be caused by the constant movement of animals, due to the practice of interstate cattle trade and also strongly suggestive for recent infections (23, 24).

The SB0295 profile was the second most prevalent *spoligotype* in this study (29%) and has been referenced in Brazil with a prevalence of 24% (23). This is similar to that in the Midwest Region of the country, being identified in 16.2% of the total isolates (25). The two isolates identified in buffaloes as SB0295 were also recorded in the Amazon region in mixed buffalo and dairy cattle breeding areas under the same management condition as reported by Carneiro et al. (26). SB0295 was identified in buffaloe isolates in Argentina, highlighting the propagation of common *M. bovis* strains among bovines and buffaloes (23).

*Spoligotype* SB0852 was identified in two isolates. According to the international database, SB0852 has only been registered in Italy (27), suggesting a process of natural selection of these strains between geographic locations (25) or convergent evolution (23).

Finally, two *spoligotypes* were observed in this study single isolates only, with the case for SB0120 being similar to the low frequency of occurrence in other regions of the country (6, 23, 28). The other was from a bovine that presented a *spoligotype* not present in the international database; this could be due to some microevolutionary events in the DR regions of a strain with an existing pattern (29).

In the region of development of the study, bovine tuberculosis is characterized as endemic, and the practice of commercialization and consumption of milk and fresh products increases the risk of zoonotic transmission, increasing the risk of sharing *M. bovis* isolates common among dairy cattle and the human population of the region, as previously recorded in other studies in different areas of the world. Genomic diversity in the *M. tuberculosis* complex remains a significant factor in the pathogenesis of tuberculosis, which can affect the virulence, transmissibility, host response, and drug resistance (29).

The genotyping performed in this study from the set of 24-*loci* MIRU-VNTR is recommended for the comparative study of *M. bovis* profiles worldwide (5). Molecular genotyping identified 13 distinct genetic profiles, suggesting a diversity of *M. bovis* within and between the regions studied and considerable higher discriminatory power as compared to *Spoligotyping*. This is according to earlier results obtained both in Brazil (25) and in other countries. This demonstrated that although a large cluster was observed by *spoligotyping* alone, there exists genetic diversity among the strains of *M. bovis* in Pernambuco, probably due to the movement of animals between different regions, states, and rural properties (23, 25).

The analysis of allelic diversity of the different MIRUs are similar to those found by Souza Filho et al. (30) and Carvalho et al. (25) and demonstrating that for this MTBC species, only six of 24 *loci* allowed good discrimination, different from *M*. *tuberculosis* (31). The HGDI of 24-MIRU-VNTR and *spoligotyping* in this study was 0.980 and 0.713, respectively, close to that observed by Carvalho et al. (25) with values 0.980 and 0.810 and the HGDI of 0.912 reported by Souza Filho et al. (30). Therefore, it seems that simultaneous consideration of both genotyping techniques for clustering might be more accurate for *M. bovis* transmission studies, also in the present study. However, the association between these techniques has been considered the best strategy for the molecular typing of *M. bovis* because they present better reproducibility and reliability, aiming at the analysis of strains mycobacterial (25).

This study is of great importance for the region as it is the first work carried out on molecular genotyping through the association between *Spoligotyping* and MIRU-VNTR aiming at the molecular characterization of *M. bovis* isolates and identification of circulating genotypes in the state of Pernambuco. The importance of *M. bovis* as a cause of human tuberculosis is worth mentioning, although sometimes neglected, especially in developing countries. The consumption of raw milk and dairy products and the constant exposure to reservoir animals are considered the main risk factors in the epidemiological chain of infection.

## Conclusion

The consumption of raw milk and dairy products is a frequent habit in the region, which, together with data on the occurrence of bovine tuberculosis, increases the risk of zoonotic transmission, alerting the possibility of sharing common *M. bovis* strains between dairy cattle and the population. The genotypic characterization allowed the identification of different *M. bovis* genotypes circulating in the state of Pernambuco, presenting both two large clusters by *spoligotyping* but evidencing considerable heterogeneity when using 24-MIRU-VNTR. Considering the diversity of genotypes obtained by combining *spoligotyping* and 24-MIRU-VNTR in the present setting, this methodology could be additive during transmission studies.

## Data Availability Statement

The original contributions presented in the study are included in the article/supplementary materials, further inquiries can be directed to the corresponding author/s.

## Ethics Statement

The animal study was reviewed and approved by Animal Use Ethics Committee (CEUA), of the Federal Rural University of Pernambuco with license no. 09/2017 CEPE/ UFRPE in accordance with COBEA and National Institute of Health Guide for Care and Use of Laboratory Animals standards.

## Author Contributions

EM, CM, JA, MF, HL, and AP: conducted and performed the microbiological diagnostic design of mycobacterial culture procedures. HG, PS, CM, and JA: constructed the molecular diagnostic methodology. EM, HG, PS, IS, ML, and RF: conducted and performed the molecular tests of genotyping and molecular typing. EM, CM, HG, and PS: accurately reviewed the manuscript. All authors have read and approved the final version of the manuscript.

## Conflict of Interest

The authors declare that the research was conducted in the absence of any commercial or financial relationships that could be construed as a potential conflict of interest.

## Publisher's Note

All claims expressed in this article are solely those of the authors and do not necessarily represent those of their affiliated organizations, or those of the publisher, the editors and the reviewers. Any product that may be evaluated in this article, or claim that may be made by its manufacturer, is not guaranteed or endorsed by the publisher.
